# Whole-Genome Sequencing and Comparative Analysis of *Yersinia pestis*, the Causative Agent of a Plague Outbreak in Northern Peru

**DOI:** 10.1128/genomeA.00249-12

**Published:** 2013-02-28

**Authors:** O. Cáceres, J. Montenegro, C. Padilla, D. Tarazona, H. Bailón, P. García, M. Céspedes, P. Valencia, H. Guio

**Affiliations:** a Laboratorio de Biotecnología y Biología Molecular, Instituto Nacional de Salud, Lima, Peru; b Laboratorio de Zoonosis Bacteriana, Instituto Nacional de Salud, Lima, Peru; c Centro Nacional de Salud Pública, Instituto Nacional de Salud, Lima, Peru; d Asociación Latinoamericana de Biotecnología (ALBIOTEC), Lima, Peru

## Abstract

The plague is a zoonotic disease caused by the bacterium *Yersinia pestis*. Here, we report the complete genome sequence of the *Y. pestis* strain INS, which was isolated from swollen lymph gland aspirate (bubo aspirate) of an infected patient from a pneumonic outbreak in 2010 in northern Peru.

## GENOME ANNOUNCEMENT

 Three biovars of *Yersinia pestis* have been characterized: Antiqua, Medievalis, and Orientalis (1). The Antiqua and Medievalis are ancient strains that were responsible for the plague of Justinian and the Black Death, respectively. Orientalis is responsible for the modern pandemic and has been recognized all around the world. The genomes of several strains of *Yersinia pestis* (CO92, KIM, and others) have been sequenced and annotated.

A total of 5 µg of DNA was sheared using a Covaris ultrasonicator, and a DNA library was prepared using the Fragment library kit according to the manufacturer’s instructions (Applied Biosystems). The DNA library was sequenced in the SOLiD 3 Plus genome analyzer, which yielded 250-fold coverage. Bioscope software (Applied Biosystems) was used to correct dibase miscalls prior to assembly. Mapping of the reads was achieved using Bowtie 2.0 (2). The *Y. pestis* strain CO92 (NCBI accession no. AL590842.1) genome sequence was used as a reference for reassembly, providing a total of 18 contigs. Consensus sequence was obtained with SAMtools (3) and Gap5 (4) for further analysis. For the genome annotation, we used Rapid Annotations using Subsystems Technology (RAST) (5), and comparative analysis was done with the fully annotated genome of strain CO92 (6). We used SNP Finder (7) for single-nucleotide polymorphisms (SNP) and small indel detection between the reference and assembled genomes.

Genome-wide comparison of strain INS to several other genomes was performed using BLAST Ring Image Generator (BRIG) (8). The plasmids pCD1 (NCBI accession no. AL117189.1), pMT1 (NCBI accession no. AL117211.1), and pPCP1 (NCBI accession no. AL109969.1) were also used as references for the reassembly of the reads that had not been mapped.

*Y. pestis* INS is 4.6 Mb long and 99.93% identical to *Y. pestis* CO92. It has an average G+C content of 47.6%. Genome annotation found 4,214 protein-coding sequences, 19 rRNA genes, and 69 tRNA genes. From these, 2,316 were assigned unequivocal functions, mainly in virulence factors, like invasins (*n* = 4), adhesion (*n* = 6), toxins (*n* = 17), invasion and intracellular resistance (*n* = 19), heme and iron uptake (*n* = 20), and resistance to antibiotics (*n* = 29). We found 63 SNPs, none of which affected protein-coding sequences of the genes that were annotated. However, 43 of these 63 SNPs might influence gene expression due to their location within the 5′ or 3′ untranslated regions (UTRs) (9). Additionally, we found three pathogenic plasmids: pCD1, pMT1, and pPCP1. The first one carries a complete type IV secretory system, which increases its capacity for cell invasion and defense neutralization. The second one holds the murine toxin (MT), which allows the bacterium to survive at 37°C. The last one has a plasminogen activator gene that helps to evade the immunological response. Moreover, pCD1 had one deletion of 1 kb and 2 SNPs. This deletion was found next to the IS*100* element, which counts for significant recombination rates through horizontal gene transfer (10). On the other hand, pMT1 had 4 deletions and 2 SNPs. It is not yet clear whether these one-gene deletions correspond to highly mobile genetic elements.

*Y. pestis* INS has similarities to La Paz strain, mainly in the presence of a multidrug translocase gene (11) that confers increased antibiotic resistance. Our findings confirm the presence of the 1.ORI group in our country and the expansion of the biovar Orientalis (12).

### Nucleotide sequence accession numbers.

This Whole Genome Shotgun project has been deposited at DDBJ/EMBL/GenBank under the accession no. AMQL00000000. The version described in this paper is the first version, AMQL01000000.
